# Evolutionary Dynamics of the W Chromosome in Caenophidian Snakes

**DOI:** 10.3390/genes9010005

**Published:** 2017-12-28

**Authors:** Barbora Augstenová, Sofia Mazzoleni, Lukáš Kratochvíl, Michail Rovatsos

**Affiliations:** Department of Ecology, Faculty of Science, Charles University, Prague, 12844, Czech Republic; augstenova.barbora@gmail.com (B.A.); sofia.mazzoleni@outlook.com (S.M.); michail.rovatsos@natur.cuni.cz (M.R.)

**Keywords:** heterochromatin, sex chromosomes, GATA, telomeres, microsatellites, FISH

## Abstract

The caenophidian (assigned also as “advanced”) snakes are traditionally viewed as a group of reptiles with a limited karyotypic variation and stable ZZ/ZW sex chromosomes. The W chromosomes of the caenophidian snakes are heterochromatic, and pioneering studies demonstrated that they are rich in repetitive elements. However, a comparative study of the evolutionary dynamics of the repetitive content of the W chromosome across the whole lineage is missing. Using molecular-cytogenetic techniques, we explored the distribution of four repetitive motifs (microsatellites GATA, GACA, AG and telomeric-like sequences), which are frequently accumulated in differentiated sex chromosomes in vertebrates, in the genomes of 13 species of the caenophidian snakes covering a wide phylogenetic spectrum of the lineage. The results demonstrate a striking variability in the morphology and the repetitive content of the W chromosomes even between closely-related species, which is in contrast to the homology and long-term stability of the gene content of the caenophidian Z chromosome. We uncovered that the tested microsatellite motifs are accumulated on the degenerated, heterochromatic W chromosomes in all tested species of the caenophidian snakes with the exception of the Javan file snake representing a basal clade. On the other hand, the presence of the accumulation of the telomeric-like sequences on the caenophidian W chromosome is evolutionary much less stable. Moreover, we demonstrated that large accumulations of telomeric-like motifs on the W chromosome contribute to sexual differences in the number of copies of the telomeric and telomeric-like repeats estimated by quantitative PCR, which might be confusing and incorrectly interpreted as sexual differences in telomere length.

## 1. Introduction

Snakes represent around one third of all current species of squamate reptiles. However, the reasons for their unusual evolutionary success are not known. Not all snake groups are equally diversified; nearly 90% of snake species (*ca*. 3000 out of 3400) belong to the group Caenophidia (advanced snakes), particularly to their subclade Colubroidea [1]. Thus, the key to understanding the evolutionary success of snakes is in uncovering the processes responsible for the superradiation of caenophidian/colubroid snakes in the last 80 million years (dating follows [2]). The colonization of new areas and the evolution of advanced venom-delivery systems were suggested to be responsible for the large diversity of caenophidian and colubroid snakes [3]. Nevertheless, it was also speculated that the enormous difference in the diversification rates between non-caenophidian and caenophidian snakes can be attributed to the differences in the degree of differentiation of sex chromosomes [4].

The snake karyotypes are usually very stable, and many the so-far studied species of non-caenophidian and caenophidian snakes possess the putative ancestral snake karyotype with 36 chromosomes (16 macro- and 20 micro-chromosomes) [5,6]. Nevertheless, only caenophidian snakes possess well-differentiated ZZ/ZW sex chromosomes with the highly heterochromatic W chromosome, which seems to be their important synapomorphy [4,7,8]. The Z chromosomes share similar gene content across 28 representative species from all caenophidian families (Acrochordidae, Colubridae, Elapidae, Homalopsidae, Lamprophiidae, Pareatidae, Viperidae, Xenodermatidae) [4], documenting the homology of sex chromosomes across this whole group. On the other hand, sex chromosomes of non-caenophidian snakes are only poorly differentiated and have not been properly identified in most lineages yet [4,9], with the important exception of the boa *Boa imperator* and the python *Python bivittatus*, which possess likely independently-evolved XX/XY sex chromosomes [10]. Most of the genes present in the caenophidian Z chromosome are lacking in their W chromosome [4,9], although certain genes are known from the W chromosome, as well [11,12]. The genes linked to the Z chromosome are evolving faster than autosomal genes [9]. Additionally, this “fast Z” effect was exactly suggested as the mechanism responsible for the faster evolution of caenophidian snakes reflected in their high diversification [4].

Tightly connected to this rapid evolution of Z-linked loci, but less studied, is the degeneration of the caenophidian W chromosome. Various oligonucleotide motifs are detected in long tandem repeats across vertebrate genomes, more commonly in heterochromatic regions such as (peri)centromeric regions and non-recombining regions of sex chromosomes [11,13]. For example, the accumulation of the (AG)_n_ motif has been reported in the Y chromosomes of the skink *Bassiana duperreyi* and the turtle *Emydura macquarii*, as well in the W chromosomes of chicken [14] and the lacertid lizard *Eremias velox* [15]. The most common motifs accumulated in sex chromosomes are likely (GATA)_n_ and (GACA)_n_. In the caenophidian snakes, both of these motifs were detected in the heterochromatic parts of the W chromosomes as a part of the larger unit, the banded krait minor-satellite DNA (*Bkm*) repeat, documented in the members of the families Viperidae, Elapidae and Colubridae [16]. Beyond the caenophidian snakes, extensive (GATA)_n_ accumulations have been reported for example in the Y chromosomes of the turtle *Chelodina longicollis*, the pygopodid gecko *Aprasia parapulchella*, the skink *Bassiana duperreyi* [14] and four species of the iguana genus *Anolis* [17]. The accumulation of the *Bkm* repeats was proposed as a pivotal event in the formation of sex-specific heterochromatin and hence differentiation of sex chromosomes [18,19]. However, the variability in the occurrence of the microsatellites on sex chromosomes across the phylogenetic spectrum probably reflects independent accumulations rather than common ancestry and questions the general involvement of the *Bkm*-like repeats in the heterochromatinization of sex chromosomes [14,15]. Recently, we found that (GATA)_n_ accumulations are not present in the highly heterochromatic W chromosome of the Javan file snake (*Acrochordus javanicus*), a member of the family Acrochordidae, while it occurs in the heterochromatic W chromosome of the dragon snake (*Xenodermus javanicus*, Xenodermatidae) [7,8]. According to the recent phylogenetic hypotheses [2,20,21], the family Acrochordidae is a sister of all colubroid snakes, and the family Xenodermatidae is a sister of all other colubroid families. Therefore, we suggested that in snake evolution, the formation of the sex-specific heterochromatin preceded the emergence of GATA accumulations on the W chromosome, which would question the role of the *Bkm* repeats in the origin of sex-specific heterochromatin even in snakes. Nevertheless, we cannot exclude the alternative scenario, i.e., the secondary loss of the *Bkm* accumulations in the ancestor of *A. javanicus.* The later hypothesis would expect that the presence of the *Bkm* accumulations on the heterochromatic W is evolutionarily less stable than assumed across caenophidian snakes.

In addition to the *Bkm* repeats, the W chromosome in the dragon snake *X. javanicus* possesses extensive amplifications of the six-nucleotide motif (TTAGGG)_n_ [7]. This motif is commonly amplified in the terminal position of vertebrate chromosomes by the cellular enzyme, telomerase, and has a crucial role in genome replication and chromosome stability (for reviews, see [22,23,24]). However, extensive amplifications of telomeric-like motifs have been documented in interstitial positions in many vertebrates, including mammals [25,26,27], fishes [28], amphibians [29,30] and reptiles [23,31,32]. Across squamate reptiles, the presence of interstitial telomeric repeats (ITRs) is a rule rather than an exception [23]. Accumulations of telomeric-like motifs are often present also in differentiated sex chromosomes as for instance in the W chromosome of the gecko *Underwoodisaurus milii* [31], the lacertid lizard *Lacerta agilis* [33] or the dragon lizard *Pogona vitticeps* [34], but they are lacking in the W chromosome of the Javan file snake [8]. 

The variability in the distribution of telomeric-like motifs, as well the differences in the size and shape of the W chromosome among species [6,35] indicate that the repetitive content of the W chromosome across caenophidian snakes might be highly variable. However, a comparative study allowing determination of its evolutionary dynamics across caenophidian snakes is missing. Here, using molecular-cytogenetic techniques, we examined the heterochromatin distribution and partial repetitive content across six families of the caenophidian snakes: Acrochordidae, Colubridae, Homalopsidae, Lamprophiidae, Viperidae and Xenodermatidae. We supplemented these original data with the literature records to reconstruct the evolutionary dynamics and to explore the stability of the presence of the tested repetitive elements in the W chromosomes across caenophidian snakes.

## 2. Materials and Methods

### 2.1. Studied Material

We studied 13 forms of caenophidian snakes: *A. javanicus* (Acrochordidae), *Elaphe bimaculata*, *Lampropeltis ruthveni*, *Lampropeltis triangulum*, *Natrix natrix*, *Pantherophis guttatus*, *Zamenis situla* (Colubridae), *Homalopsis buccata* (Homalopsidae), *Boaedon* sp. 1 (brown colored, morphologically resembling *Boaedon fuliginosus*), *Boaedon* sp. 2 (black colored, morphologically resembling *Boaedon olivaceus*), *Boaedon* sp. 3 (green colored, morphologically resembling *Boaedon olivaceus*) (Lamprophiidae), *Crotalus durissus* unicolor (Viperidae) and *X. javanicus* (Xenodermatidae) (Table S1). The studied animals originated from the pet trade. We supplemented our dataset by the additional data on the repetitive content in the previously-studied species *A. javanicus* [8], *X. javanicus* [7], *Protobothrops flavoviridis* [11,36], *Notechis scutatus* [14,19,37,38], *Elaphe quadrivirgata* [14,39,40] and *Rhabdophis tigrinus* [14,39].

In the specimens we studied, we collected blood and used it for whole blood cell cultures and DNA isolation. The blood taking was approved by the Central Commission for Animal Welfare of the Czech Republic (Protocol No. 35484/2015-16). The genus *Boaedon* is likely a complicated species complex containing several undescribed taxa with not yet settled taxonomy [41,42]. Therefore, we amplified and sequenced mitochondrial DNA fragments from all studied specimens of the genus in order to “DNA barcode” our studied material for future proper determination. For genomic DNA isolation, we used the DNeasy Blood and Tissue Kit (Qiagen, Valencia, CA, USA). We used the reptile-specific primers RepCOI-F/RepCOI-R for amplifying the standard barcoding region of mitochondrial gene *cytochrome oxidase* subunit I (COXI) [43]. The PCR reaction protocol and amplification conditions were reported previously [44]. In addition, we sequenced a fragment of the mitochondrial gene *cytochrome b* (cytb) using the universal primers H16064/L14910 [45]. The amplification conditions were described previously [41,42]. The PCR products were sent for bi-directional sequencing to Macrogen (Seoul, Korea). The sequences were aligned using BioEdit v5.0.9 [46] and subsequently analyzed in MEGA v6.0.5 [47] and DnaSP v5.10.1 [48]. A BLAST search was performed to compare our sequences with those from previous studies [41,42].

### 2.2. Chromosome Preparations and Staining

We used whole blood cell cultures for the preparation of metaphase chromosome spreads following the protocol described in [31]. The chromosomal preparations were stained by Giemsa. The C-banding was used for visualization of the constitutive heterochromatin and detection of heterochromatic sex chromosomes following [49], with a slight modification, as the chromosomes were counterstained by 4′,6-diamidino-2-phenylindole (DAPI) instead of Giemsa.

### 2.3. Fluorescence In Situ Hybridization with Telomeric Probe

Fluorescence in situ hybridization (FISH) with the telomeric probe was used to visualize the distribution of the telomeric-like motif (TTAGGG)_n_. The probe was prepared by PCR using the primers (TTAGGG)_5_ and (CCCTAA)_5_ without genomic DNA template [50] and labelled in the same PCR reaction with dUTP-biotin. The PCR reaction mix and amplification conditions are presented in detail in [23]. The PCR product was precipitated and resuspended in 300 μL of hybridization buffer (50% formamide/2× saline-sodium citrate buffer, SSC) and stored in the freezer (−20 °C). Prior to hybridization, the probe was denatured at 73 °C for 6 min and chilled temporally on ice until use. The slides with chromosomal material were treated with RNAse and pepsin, fixed with 4% formaldehyde and then dehydrated through a series of 70%, 85% and 100% ethanol. They were subsequently denatured in 70% formamide/2× SSC at 75 °C for 4 min and dehydrated. We applied 11 μL of the telomeric probe per slide and incubated the slide overnight at 37 °C. The following day, the slides were washed three times in 50% formamide/2× SSC at 42 °C and in 2× SSC. We incubated the slides with 100 μL of 4× SSC/5% blocking reagent (Roche, Basel, Switzerland) at 37 °C for 45 min. For telomere detection, we used a modified avidin-FITC/biotinylated anti-avidin system (Vector Laboratories Burlingame, CA, USA) for an amplification of the fluorescent signals (for the detailed protocol, see [23]). Slides were stained with DAPI and mounted with the antifade medium Fluoroshield (Sigma-Aldrich, St. Louis, MO, USA).

### 2.4. Fluorescence In Situ Hybridization with Microsatellites

We used microsatellite probes (GATA)_8_, (GACA)_8_ and (AG)_15_ to explore the accumulation of these microsatellite motifs in the W chromosome. The procedure was similar to FISH with the telomeric probe with slight differences in probe preparation and post-hybridization washes. The microsatellite probes were synthesized and 5′-end-labelled with biotin by Macrogen (Korea). Then, 30 pmol of microsatellite probe were resuspended in 11 μL hybridization buffer (50% formamide, 2× SSC, 10% sodium dodecyl sulfate, 10% dextran sulfate, 1× Denhard’s buffer, pH 7). The post-hybridization washes were performed in 0.4× SSC/0.3% Nonidet P-40 (Sigma-Aldrich, St. Louis, MO, USA) at 40 °C for 2 min and in 2× SSC/0.1% Nonidet P-40 at room temperature for 30 s.

### 2.5. Microscopy and Image Analyses

The photos of metaphases were captured by the Provis AX70 (Olympus, Tokyo, Japan) fluorescence microscope equipped with a DP30BW digital camera (Olympus). The photos from FISH experiments were further processed using the DP manager imaging software (Olympus, Tokyo, Japan). Karyograms were constructed from Giemsa-stained preparations in the Ikaros karyotyping software (Metasystems, Altlussheim, Germany).

### 2.6. Estimation of Sexual Differences in the Number of Telomeric-Like Repeats

The accumulation of the telomeric-like repeats in the W chromosome should lead to the differences in repeats of telomeric-like sequences between sexes. Their number can be quantified by the quantitative polymerase chain reaction (qPCR) [31].

For the amplification of the telomeric-like sequences, we used the primers by [51]. In addition, we used the gene *MDS1 and EVI1 complex locus* (*mecom*) for normalization of the quantification cycle values (crossing point, Cp). The primers specific for the gene *mecom* [4] and the telomeric motifs [51], as well as the amplification conditions were described previously [4]. The qPCR was performed using the LightCycler II 480 (Roche Diagnostics, Basel, Switzerland). The samples were run in triplicate in a 15-μL reaction volume, containing 0.2 ng of genomic DNA, 7.5 μL SYBR Premix Ex Taq II (Takara Bio, Kusatsu, Japan) and 0.3 μL of each primer (10 pmol/μL stock solution). An estimation of the amount of the telomeric motifs was calculated from Cp values and was subsequently normalized to the dose of the reference gene *mecom* from the same DNA sample (for the formulas, see [52]). Subsequently, we calculated the ratio of the relative numbers of copies of the telomeric-like repeats between sexes. A ratio around 1.0 is expected in species without ITRs on the W chromosome, while a much higher ratio is expected in species with the accumulation of telomeric-like sequences on the W chromosome. Our estimation of the W-linked ITRs’ variation assumes that telomeres of the Z and W chromosomes are comparable in size and that there are no ITRs on the Z chromosomes. Moreover, it neglects that the number of telomeric motifs can vary between conspecific individuals due to an individual polymorphism typical for any repetitive sequences and also due to shortening of telomere with age [53]. Therefore, our approach is only a rough estimation of the size of the W-linked ITRs, but still, we expect that it should correspond to the results of the observation of ITRs in sex chromosomes from our FISH experiments. 

## 3. Results

### 3.1. Species Identification in the Genus *Boaedon*

The sequences of *cytb* and COXI were successfully amplified in all our specimens of the genus *Boaedon* and compared with the sequences in GenBank [41,42] revealing three distinct haplotypes. The forms assigned as *Boaedon* sp. 2 and *Boaedon* sp. 3 have a genetic p-distance of 10.7% and 11.4%, respectively, in comparison to *Boaedon* sp. 1, and 8.9% to each other. Therefore, we assume that all three *Boaedon* forms correspond to distinct species. However, due to the unresolved taxonomy in this group of snakes (see [41,42]), we cannot safely assigned any valid species names to them. However, due to our DNA barcoding, the karyotype descriptions of our samples will be possible to attribute to species names in the future, after a careful taxonomic re-evaluation of this species complex. All haplotypes are deposited in GenBank.

### 3.2. Karyotypes of Previously Cytogenetically Unstudied Species

To our knowledge, the karyotypes of *L. ruthveni*, *L. triangulum*, *Z. situla* and our forms of the genus *Boaedon* have not been previously described. The karyotype of *E. bimaculata* will be described elsewhere. *L. ruthveni*, *L. triangulum* and *Z. situla* have similar karyotypes with 2n = 36 chromosomes; sex chromosomes are the fourth pair of the complement, which is frequent among caenophidian snakes [6]. The genus *Boaedon* exhibits unexpected variability in the karyotype. The karyotype of *Boaedon* sp. 1 consists of 42 chromosomes, that of *Boaedon* sp. 2 of 40 chromosomes and that of *Boaedon* sp. 3 of 34 chromosomes. The karyotype with 2n = 34 chromosomes was earlier described for *B. fuliginosus* [54]. The sex chromosomes are the fourth pair of the complement in *Boaedon* sp.2, but the first pair of the complement in *Boaedon* sp. 1 and *Boaedon* sp. 3. 

Karyograms of all previously unstudied species are depicted in the Supplementary Material (Figure S1). 

### 3.3. Morphology of W Chromosome and Distribution of Constitutive Heterochromatin

**Figure 1:**
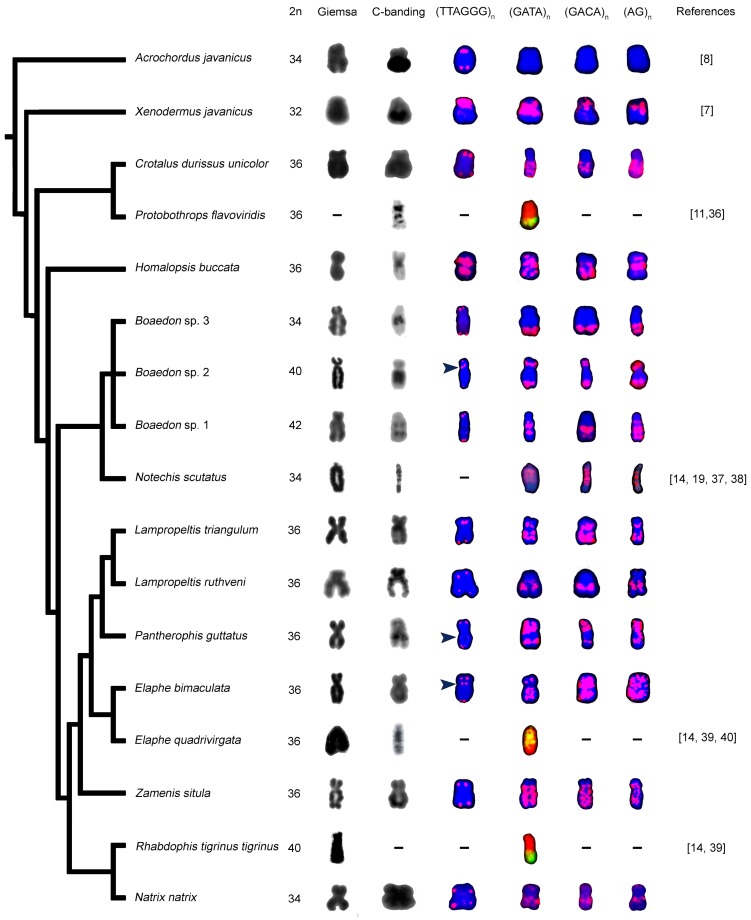
Comparative phylogenetic reconstruction of the heterochromatic content of the W chromosomes in Caenophidia. The phylogenetic tree of studied snakes was completed according to phylogenies by [2]. We compared the morphology of W chromosomes (Giemsa stain), the pattern of heterochromatin accumulation (C-banding) and the topology of four microsatellite repeats: (TTAGGG)_n_, (GATA)_n_, (GACA)_n_ and (AG)_n_ (FISH). The chromosomes from C-banding and FISH experiments were counterstained by 4′,6-diamidino-2-phenylindole (DAPI). Photos of C-banding were inverted. In the case of FISH experiments, the chromosomes with DAPI were stained blue, and the fluorescence signal of probes was pseudo-colorized in red. Dashes represent missing data. Interstitial telomeric repeats (ITRs) on W chromosomes are indicated by arrows when they are not easily visible. For data collected from the bibliography, see the references inside the figure.

The W chromosome is subtelocentric or submetacentric in almost all studied species of the caenophidian snakes (Figure 1). We used C-banding to visualize constitutive heterochromatin. The heterochromatic regions on the W chromosome were present in all caenophidian species studied by us. The species differ in the size of the W-linked heterochromatic blocks (Figure 1). The heterochromatic blocks cover the whole W chromosome in *C*. *durissus unicolor, E. bimaculata*, *L. triangulum*, *L. ruthveni*, *N. natrix* and *Z. situla*, but only part of it in *H. buccata*, *P. guttatus* and all three *Boaedon* lineages (Figure 1). Wherever identifiable, the Z chromosomes do not include heterochromatic blocks, ITRs or accumulations of tested repetitive motifs.

### 3.4. Distribution of Telomeric-Like Sequence (TTAGGG)_n_ in W Chromosomes

The telomeric repeats were detected in the terminal position of all chromosomes, including sex chromosomes in all studied species. ITRs were detected in the W chromosomes of *E. bimaculata*, *H. buccata*, *P. guttatus* and in *Boaedon* sp. 2. In *X. javanicus*, the higher accumulation of ITRs is present around the centromeric region of the W chromosome [7].

**Figure 2:**
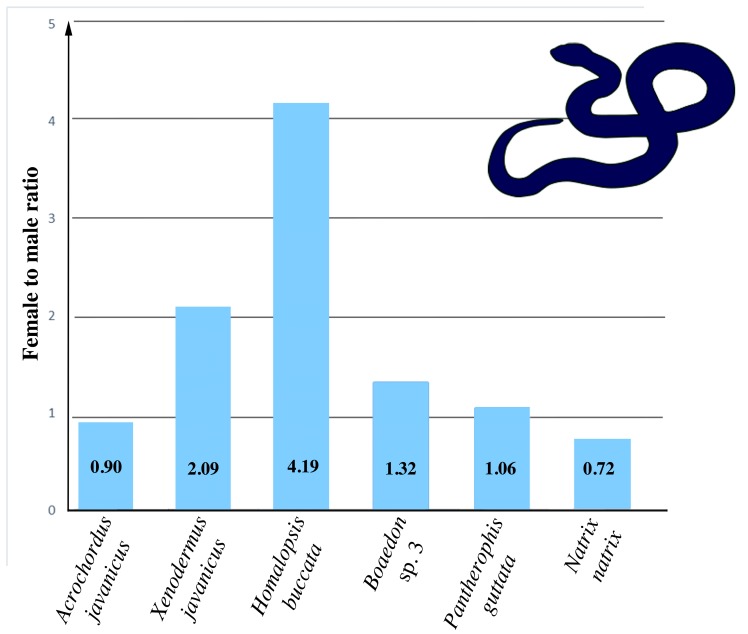
Female-to-male ratios of the number of telomeric-like motifs.

The presence of the telomeric-like motif accumulation on the W chromosome was subsequently confirmed by the measurements of the relative number of the telomeric-like repeats by qPCR (Figure 2). Almost equal numbers of the telomeric-like repeats between sexes were estimated by qPCR in *A. javanicus*, *Boaedon* sp. 3 and *N. natrix*, i.e., in the three species where FISH with the telomeric probe did not reveal any ITRs in the W chromosomes (Figure 1). In addition, we did not detect any notable sex-specific difference in the amount of telomeric-like repeats in *P. guttatus*, which possesses rather small ITRs in the centromeric region of the W chromosome. In contrast, the significant differences between sexes were measured by qPCR in *H. buccata* and *X. javanicus* (see Figure 2), the species with extensive blocks of ITRs in their W chromosomes clearly visible in the results of the FISH with the telomeric probe (Figure 1).

### 3.5. Accumulation of Microsatellites on W Chromosomes

We detected extensive accumulations of the repetitive microsatellite motifs (GATA)_n_, (GACA)_n_ and (AG)_n_ on the W chromosomes in all herein studied species. However, the position and extent of the accumulations of the microsatellite motifs differ significantly, even in closely-related species (Figure 1). 

## 4. Discussion

We can conclude that the distribution of the heterochromatin and the repetitive content of the W chromosome are highly variable among caenophidian snakes. The distribution of the accumulations of particular repetitive sequences in the W chromosome, as well as the presence of ITRs can be different even in closely-related species such as within the genus *Boaedon* (Figure 1). This supports the view that differentiated and degenerated sex chromosomes are among the most evolutionary dynamic regions of the genome [14,15,55].

On the other hand, certain aspects of the W chromosomes across caenophidian snakes are remarkably stable. For example, we detected a heterochromatic region in the W chromosomes in every studied caenophidian snake (Figure 1), although heterochromatin seems to disappear completely from the degenerated Y chromosomes in certain lineages of iguanas, the other reptile lineage with a long-term stability of differentiated sex chromosomes [56]. Furthermore, up to now, all tested species of colubroid snakes, but not the single tested species of their sister lineage, the family Acrochordidae, possess the notable accumulations of the repetitive microsatellite motifs GATA, GACA and AG in their W chromosomes (Figure 1). Such stability in the presence of the accumulations of these motifs across colubroid snakes and the lack of the accumulation of microsatellite motifs in homomorphic sex chromosomes of the python *Liasis fuscus* [19] and other non-caenophidian snakes [57] suggest that the situation in the acrochordid represents a primitive state rather than a secondary loss of these accumulations. The presence of these accumulations is stable across a large colubroid radiation, which suggests that their loss is rare. It would be a strange coincidence if a rare loss not detected by us within Colubroidea happened just in the sister lineage of colubroid snakes. If the scenario of the emergence of W-linked heterochromatin in the common ancestor of the caenophidian snakes, but the emergence of the *Bkm*-repeats including GATA and GACA motifs only later in the evolution in the common ancestor of the colubroid snakes is correct, the *Bkm* repeats were not able to play an important role in the formation of sex heterochromatin in snakes as previously suggested [58]. It is also of interest that the distributions of the GATA and GACA accumulations on the snake W chromosomes slightly differ in some snake species (Figure 1), although they are supposed to be a part of the same repetitive element (*Bkm*). It is possible that these motifs occur also in other repetitive elements accumulated on the snake W chromosomes.

We demonstrated that the accumulations of the telomeric-like repeats on the W chromosomes occur relatively frequently among colubroid snakes (Figure 1). Their phylogenetic distribution suggests that they might evolve independently several times within Colubroidea as they did also in other squamate lineages [31,33,34]. These accumulations highly differ in size (Figure 1), and the most pronounced of them are easily measurable by qPCR detecting copy numbers of telomeric-like repeats in genomes. Interestingly, qPCR is commonly used for measurements of telomere length, although it cannot differentiate between true telomeric sequences and telomeric-like sequences producing ITRs [31]. A pronounced accumulation of telomeric-like repeats linked to non-recombining regions of a sex chromosome can lead to the finding of sex-specific differences in the copy number of telomeric-like repeats in qPCR, which can be interpreted as a sexual difference in telomere length. Sexual differences in telomere length were attributed to sexual differences in life-history including aging and sex-specific ecological or reproductive roles (e.g., [59] in a colubrid snake). Our study warns against premature adaptive explanations of sexual differences in estimated telomere lengths. We suggest that a careful examination of the genomic distribution of telomeric-like repeats in a studied species should be done before any interpretation of the causes of the variability in measured telomere lengths among sexes or individuals. It seems to us that it is very difficult to compare for example telomere shortening/aging by qPCR using mixed-sex samples and to interpret sexual differences in the measured copy numbers of telomeric or telomeric-like sequence in species with sex chromosomes.

## Supplementary Materials

The following are available online at http://www.mdpi.com/2073-4425/9/1/5/s1. Table S1: Summary of species and number of examined individuals used in the current study. Figure S1: Giemsa-stained karyotypes and C-banded metaphase spreads of unstudied species from female specimens of *Boaedon* sp.1 (a,b), *Boaedon* sp.2 (c,d), *Boaedon* sp.3 (e,f), *Lampropeltis ruthveni* (g,h), *Lampropeltis triangulum* (i,j) and *Zamenis situla* (k,l).
