# Supplementary material for: Evolutionary Dynamics of the W Chromosome in Caenophidian Snakes
Source: Genes (Basel). 2017 Dec 28;9(1):5. doi: 10.3390/genes9010005 (PMC5793158; doi:10.3390/genes9010005)
Supplement: Supplementary file 1 [file genes-09-00005-s001.pdf]

Supplementary material

Table S1. Summary of species and number of examined individuals used in the current study.

Species	Family	Number of individuals	
		♂	♀
Acrochordus javanicus	Acrochordidae	1	3
Elaphe bimaculata	Colubridae		2
Lampropeltis ruthveni		1	1
Lampropeltis triangulum			1
Natrix natrix		1	1
Pantherophis guttatus			2
Zamenis situla			1
Homalopsis buccata	Homalopsidae	1	1
Boaedon sp. 1	Lamprophiidae	1	4
Boaedon sp. 2		1	2
Boaedon sp. 3		1	1
Crotalus durissus unicolor	Viperidae		1
Xenodermus javanicus	Xenodermatidae	1	1

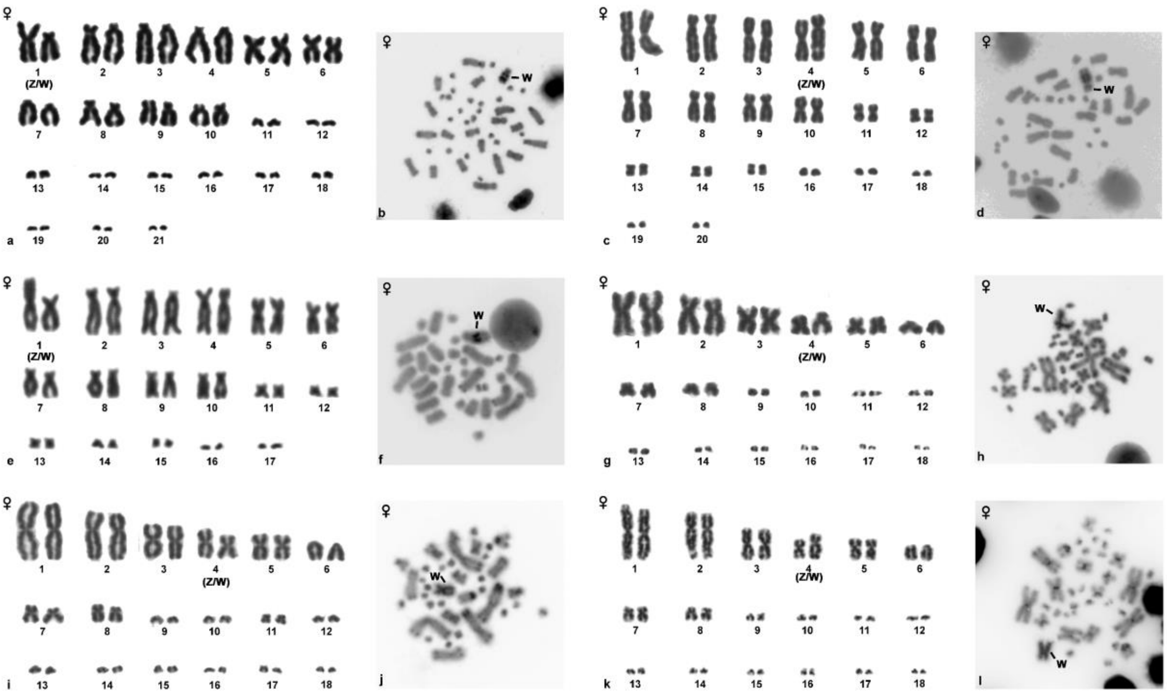

Figure S1. Giemsa stained karyotypes and C-banded metaphase spreads of unstudied species from female specimens of *Boaedon* sp.1 (a,b), *Boaedon* sp.2 (c,d), *Boaedon* sp.3 (e,f), *Lampropeltis ruthveni* (g,h), *Lampropeltis triangulum* (i,j) and *Zamenis situla* (k,l).
